# Effect of Gsk3 inhibitor CHIR99021 on aneuploidy levels in rat embryonic stem cells

**DOI:** 10.1007/s11626-014-9734-5

**Published:** 2014-02-12

**Authors:** Anagha S. Bock, Nathan D. Leigh, Elizabeth C. Bryda

**Affiliations:** 1 Rat Resource and Research Center, Department of Veterinary Pathobiology, College of Veterinary Medicine, University of Missouri, 4011 Discovery Drive, Columbia, MO 65201 USA; 2 Mass Spectrometry Facility, Department of Chemistry, University of Missouri, Columbia, MO 65203 USA

**Keywords:** Rat embryonic stem cells, CHIR99021 inhibitor, Gsk3, Aneuploidy, ES cell culture

## Abstract

Germline competent embryonic stem (ES) cells can serve as a tool to create genetically engineered rat strains used to elucidate gene function or provide disease models. In optimum culture conditions, ES cells are able to retain their pluripotent state. The type of components present and their concentration in ES cell culture media greatly influences characteristics of ES cells including the ability to maintain the cells in a pluripotent state. We routinely use 2i media containing inhibitors CHIR99021 and PD0325901 to culture rat ES cells. CHIR99021 specifically inhibits the Gsk3β pathway. We have found that the vendor source of CHIR99021 has a measurable influence on the level of aneuploidy seen over time as rat ES cells are passaged. Karyotyping of three different rat ES cell lines passaged multiple times showed increased aneuploidy when CHIR99021 from source B was used. Mass spectrometry analysis of this inhibitor showed the presence of unexpected synthetic small molecules, which might directly or indirectly cause increases in chromosome instability. Identifying these molecules could further understanding of their influence on chromosome stability and indicate how to improve synthesis of this media component to prevent deleterious effects in culture.

## Introduction

Embryonic stem (ES) cells have the ability for continuous self-renewal which requires the maintenance of a pluripotent state through unique transcriptional control (Chen and Daley 2008). The pluripotency of ES cells along with the convenience with which they can be manipulated genetically offers a powerful tool to elucidate gene function or to create animal models of disease (Li et al. 2008). The rat is a preferred animal model in many fields of biomedical research because in part, its larger size offers advantages over mice for physiological and pharmacological studies (Abbott 2004). However, it has lagged behind the mouse as a model organism because until recently, the ability to genetically manipulate the rat genome was limited (Li et al. 2008). With the establishment of germline competent rat ES cells (Buehr et al. 2008; Li et al. 2008; Voigt and Serikawa 2009), it is now possible to use the same ES cell-based techniques that have been so successful in creating genetically altered mice to create rat models.

Germline competence of ES cells is a crucial characteristic in the creation of genetically engineered animals. The standard procedure to achieve germline transmission of ES cells includes injection of ES cells into host blastocysts to make chimeric animals which are then bred to produce offsprings that are evaluated for the presence of the genetic contribution from the ES cell line. Aneuploid ES cells have very low rates of germline transmission (Longo et al. 1997). Thus, it becomes important to have a significant number of euploid cells during blastocyst injection to achieve efficient levels of germline transmission.

ES cell culture is done routinely using specific growth media which help ES cells to multiply in number while retaining pluripotency. Media components influence the characteristics of ES cells, e.g., inhibitors affect specific pathways and keep the cells in a pluripotent state. Rat ES cells are propagated using 2i media which contains two inhibitors: a MEK inhibitor, PD0325901, and the Gsk3 inhibitor, CHIR99021. The MEK/ERK signaling pathway plays a crucial role in the self-renewing state of ES cells; PD0325901 inhibitor suppresses ERK activation, thereby inhibiting ES cell differentiation (Buehr et al. 2008; Ying et al. 2008). Inhibition of Gsk3 helps ES cells to retain ground state via overall reduction of biosynthetic capacity and stabilization of β-catenin which results in stimulation of canonical Wnt signaling (Anton et al. 2007; Buehr et al. 2008; Silva and Smith 2008; Meek et al. 2013). Inclusion of these inhibitors in rat ES cell media is crucial, and therefore, the quality and concentration of these inhibitors are important parameters to control for successful rat ES cell culture and propagation.

In the course of routine expansion of rat ES cells for archiving cryopreserved stocks, we noted an unexpected increase in aneuploidy in ES cells passaged over time. This increased aneuploidy was linked to one component of the media, the CHIR99021 inhibitor, and it was dependent on the vendor source of this reagent. Further analysis suggests that the presence of other synthetic molecules in the reagent have a detrimental effect on chromosome stability.

## Materials and Methods

### *Rat ES cell lines and culture procedures.*

Rat ES cell lines SD.Tg.EC1 (RRRC# 561), F344.Tg.EC4011 (RRRC# 654) and DA-EC8 (RRRC# 464) were obtained from the Rat Resource and Research Center (University of Missouri, Columbia, MO; www.rrrc.us). All three rat ES cell lines were cultured using serum-free N2B27 media prepared as described in Ying et al. 2008. CHIR99021 inhibitor was purchased from Stemgent, Cambridge, MA (source A) or BioVision, San Francisco, CA (source B). 2i media was prepared by addition of final concentrations of 3 μM CHIR99021 from either source A or B and 0.5 μM of PD0325901 (Stemgent) to N2B27 media.

A vial of 1 × 10^6^ ES cells was brought out of cryopreservation and divided equally to seed two cultures which were identical except for the source of CHIR99021 in the 2i media. ES cells were cultured at 37°C in a CO_2_ incubator maintaining 5% humidity. The feeder cells were mitotically inactive CF-1 MEFs (Millipore, Billerica, MA) cultured with feeder media containing 10% FBS (Hyclone, Waltham, MA) and GMEM (Invitrogen, Carlsbad, CA). Since rat ES cells loosely attach to feeders, ES cells were detached from the feeders by mechanical dissociation with gentle pipetting. Rat ES cells were passaged every 48 h using Accutase (Sigma, St. Louis, MO) to obtain single cell suspension after harvesting by centrifugation. The cells were passaged at least three times after recovery from cryopreservation and harvested when they reached 60–70% confluency before preparing metaphase spreads.

### *Metaphase spread preparation and Giemsa-trypsin banding.*

Rat ES cells were cultured in 60 mm culture dishes for at least three passages and plated at a 1–2 million cell density on a 60-mm dish after the third passage. Approximately 24 h after passaging, cells were fed with 2 ml fresh 2i media and treated with 10 μg/ml of Colcemid (Irvine Scientific, Santa Ana, CA) for 1.5 h at 37°C. Cells were then dissociated into single cell suspension with Accutase and centrifuged at 150×*g* for 8 min in a 15-ml conical tube to pellet the cells. The pellet was resuspended in 4–5 ml of hypotonic solution (0.56% KCl) and incubated at room temperature for 30 min. A few drops of freshly made fixative consisting of 3:1 methanol:acetic acid (Fisher Scientific, Pittsburgh, PA) were added and mixed by inversion. The cells were centrifuged at 150×*g* for 8 min to pellet the cells. The fixation step was repeated twice before dropping the cells on wet microscope slides to prepare metaphase spreads. One- or 2-d-old slides were used for Giemsa-trypsin banding. On the day of Giemsa banding, slides were incubated in 2X SSC at 62°C and then cooled down under tap water. Slides were rinsed in 0.85% NaCl solution and then treated with 0.025% trypsin (Millipore) in 0.85% NaCl for 6–8 s and then immediately washed in 1X PBS. After dipping the slides in Gurr’s buffer (VWR, Radnor, PA), slides were stained for 7–8 min in Giemsa stain (Gibco, Carlsbad, CA). Slides were then visualized, and chromosomes were analyzed for abnormalities using ASI imaging and analysis software (Applied Spectral Imaging, Carlsbad, CA). At least 20 cells were examined for each treatment group. The distribution of differences in euploidy levels in cells cultured with media containing either source A or source B inhibitor was analyzed by Mixed Logistic Model with one factor.

### *LCMS analysis for CHIR99021 inhibitor.*

LCMS analysis on CHIR99021 inhibitor from source A and source B was performed at the Mass Spectrometry Facility at the University of Missouri, Department of Chemistry. The LC system consisted of a Thermo-Finnigan P4000 quaternary LC pump and SCM1000 vacuum degasser, an AS3000 autosampler, and a UV6000LP diode-array detector. The LC system was connected to a Thermo-Finnigan TSQ7000 triple quadrupole mass spectrometer equipped with an atmospheric pressure chemical ionization (APCI) source. Separation of sample components was accomplished using water (eluent “A”) and acetonitrile (eluent “B”) with a gradient as follows: 0 min, 1% “B”; 30 min, 100% “B”; 34 min, 100% “B”; 35 min, 1% “B”; and 40 min, 1% “B”. Sample injections were 20 μL, and samples were separated on a 4.6 × 250mm Prodigy ODS(3) column with 5 μm particles (Phenomenex, Torrance, CA). The diode array detector acquired spectra from 190 to 450 nm at a rate of 1 Hz. For the mass spectrometer, the APCI vaporizer temperature was set at 400°C, and the discharge current was 5 μA; the heated capillary was maintained at 250°C. Nitrogen was supplied to the source from the headspace of a Dewar of liquid nitrogen; the sheath gas pressure was 80 psi and the auxiliary gas flow rate was 40 (arbitrary units). Other settings were as determined during the periodic tuning and calibration of the instrument. Spectra were acquired every second across a scan range of *m*/*z* 100 to 800.

The same LCMS system and conditions were used for MS/MS experiments, with the collision cell having 1 mtorr Ar and the collision energy set at 25 eV. Only single-charged precursor ions were observed; these were selected for MS/MS in the retention time windows in which they occurred. Additional MS/MS experiments were performed by direct infusion on a Thermo LCQ DecaXP Plus ion trap mass spectrometer equipped with an APCI ion source.

## Results

### *SD.Tg.EC1 rat ES cells cultured in media with source B inhibitor showed increased levels of aneuploidy.*

**Figure 1.**
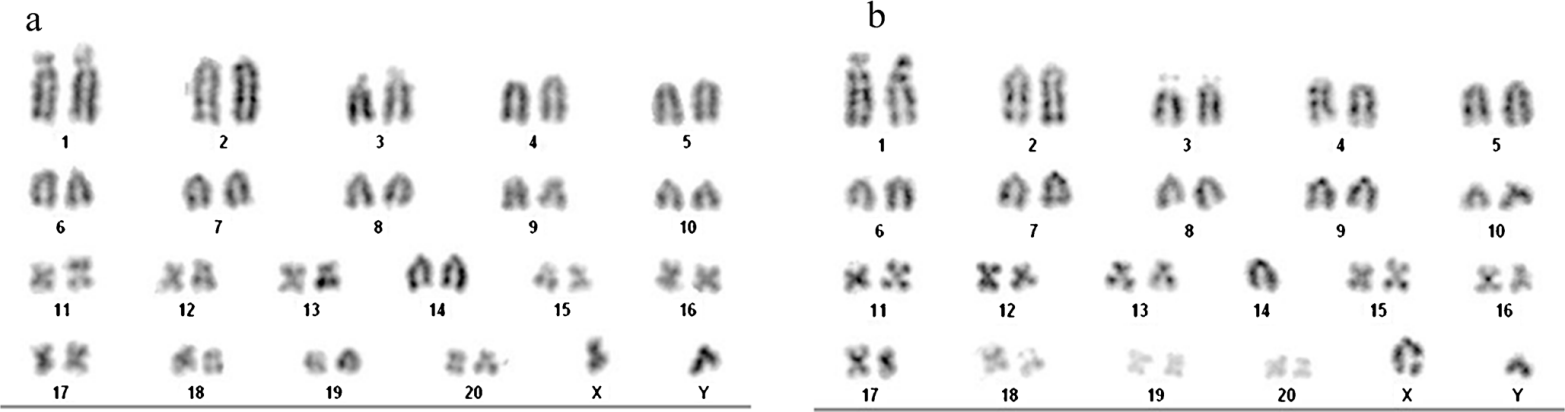
Karyotype analysis. SD-Tg.EC1, p16 metaphase chromosomes were stained with Giemsa-trypsin. **a** Cells cultured in media containing source A CHIR99021 have a normal karyotype (42, XY). **b** Cells cultured in media containing source B CHIR99021 have an abnormal karyotype (41, XY, −14). *n* = 20 metaphase spreads analyzed per culture.

**Figure 2.**
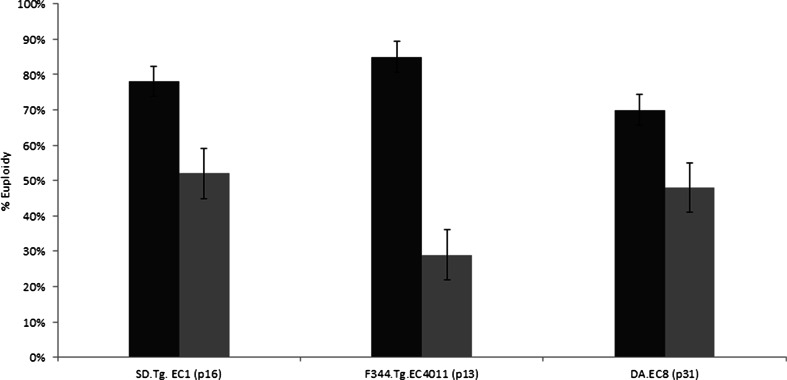
Percent of euploidy in three separate rat ES cell lines cultured with media containing either source A (*black*) or source B (*gray*) CHIR99021 inhibitor. The probability of having a normal chromosome number in cells treated with culture media containing source A is significantly higher than for cells treated with culture media containing source B for all three cell lines (*p* < 0.0001).

During routine expansion of the SD.Tg.EC1 ES cell line from a cryopreserved, low passage (p9) stock, it was noted that the cell line, which had a normal karyotype previously (>90% normal, data not shown), was now showing higher than expected levels of aneuploidy, including monosomy for Chromosome 14. A review of our lab protocol and procedures indicated that the only change that had occurred since the initial expansion and cryopreservation of the low passage SD.Tg.EC1 ES cell line was a change in vendor source for the CHIR99021 inhibitor. Based on the manufacturers’ information, the inhibitor from sources A (original source of inhibitor) and B (new vendor source) has comparable composition and purity levels. We cultured SD.Tg.EC1 cells under identical conditions except for the source of the inhibitor added to the media. Karyotype analysis of p16 cells (Fig. 1) indicated less euploidy in the cells cultured in media with source B inhibitor (52% cells with normal karyotype) than those cultured in media containing source A inhibitor (78% cells with normal karyotype) (Fig. 2). These observations suggest that the presence of source B inhibitor in ES cell media results in increased levels of aneuploidy in SD.Tg.EC1 cultured cells.

### *Use of CHIR99021 inhibitor from source A and source B shows major difference in aneuploidy levels in different rat ES cell lines.*

To investigate whether the observation of increased aneuploidy was unique to the SD.Tg.EC1 cell line, two different rat ES cell lines, F344.Tg.EC4011 (p13, 100% normal karyotype) and DA-EC8 (p31, 72% normal karyotype) were tested. The cell lines are derived from different inbred rat genetic backgrounds (Fischer 344 and Dark Agouti, respectively) as compared to the SD.Tg.EC1 cell line which has an outbred Sprague Dawley genetic background. In this set of experiments, a single vial of cryopreserved cells was evenly divided between two cultures containing 2i culture media. The sole difference between the two cultures was the source of the CHIR99021 inhibitor. Cells were passaged every 48 h for three passages after recovery from cryopreservation and karyotyping with Giemsa-trypsin banding was performed after the third passage. We did not note any overtly obvious differences in growth, cell numbers, or see evidence of differences in cell death between source A or source B cultures. However, we did find that there was a major difference in the percent of cells examined with normal versus abnormal karyotypes depending on whether source A or source B inhibitor was used (Fig. 2). The percentage of euploid cells was 85% (source A) and 29% (source B) for F344-Tg.EC4011 and 70% (source A) and 48% (source B) for DA-EC8 (Fig. 2).

For all three lines (SD.Tg.EC1, F344.Tg.EC4011, and DA-EC8), cells cultured in media containing source B inhibitor had more cells with an abnormal karyotype, specifically loss or gain of chromosomes, than the cells grown in media containing source A inhibitor. No cases of polyploidy were observed in any of the cultures examined. In Table 1, the total number of cells examined from each culture grown in media with sources A or B inhibitor is indicated, and the chromosome abnormalities are listed along with the number of cells that exhibited that particular defective chromosome makeup. DA-EC8 (p31) had overall higher levels of aneuploidy but was also at a higher passage number when analyzed.

**Table 1:** Chromosomal abnormalities observed in cultures

F344.Tg.EC4011 (p13)		DA-EC8 (p31)		SD.Tg.EC1 (p16)	
Source A (*n* = 26)	Source B (*n* = 21)	Source A (*n* = 23)	Source B (*n* = 29)	Source A (*n* = 41)	Source B (*n* = 36)
−13 (*n* = 1)	−13 (*n* = 7)	−13 (*n* = 1)	−5 (*n* = 1)	−14 (*n* = 5)	−14 (12)
−19 (*n* = 2)	−10, 13 (*n* = 1)	−19 (*n* = 1)	−13 (*n* = 4)	−10, 14 (*n* = 1)	−9, 14 (*n* = 1)
−8, 10, 14 (*n* = 1)	−13, 15 (*n* = 3)	−10, 19 (*n* = 1)	−15 (*n* = 2)	+9 (*n* = 2)	+15 (*n* = 1)
	−8, 14 (*n* = 1)	−5, 13, 15 (*n* = 1)	−5, 15 (*n* = 1)	+19 (*n* = 1)	+19 (*n* = 3)
	−8, 12,13 (*n* = 1)	−10, 13, 19 (*n* = 1)	−13, 19 (*n* = 1)		
	+18 (*n* = 2)	+15 (*n* = 1)	−8, 13, 15 (*n* = 1)		
		+19 (*n* = 1)	−2, 13, 15, 19 (*n* = 1)		
			−5, 6, 13, 16, 20 (*n* = 1)		
			+19 (*n* = 2)		
			+9, 16 (*n* = 1)		

### *Liquid chromatography mass spectrometry analysis of source A and source B CHIR99021.*

**Figure 3.**
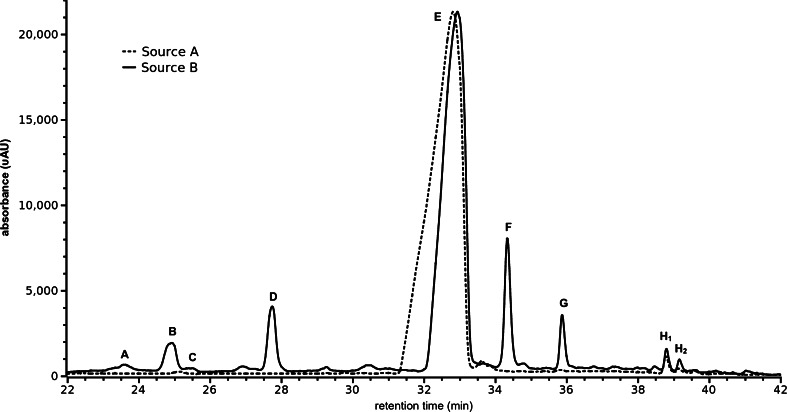
Chromatograms (*λ* = 340 nm) for samples from sources A and B; outside the retention times shown here, only the background signal is observed. The chromatogram for source B has been scaled so that the major peak approximates the magnitude of the same peak for source A. Peak labels correspond to the data in Table 2.

CHIR99021 from source B was ≥95% pure according to the datasheet provided and it was assessed for purity with HPLC. CHIR99021 from source A was greater than 95% pure according to HPLC, and its correct mass was determined by mass spectrometry according to the vendor source. In order to understand the composition of these inhibitors, the compounds were analyzed by liquid chromatography-mass spectrometry (LCMS) technique which showed differences in the source A and source B compounds. Both the compounds clearly contained inhibitor molecule (species E) (Fig. 3). Source A inhibitor showed the presence of the main inhibitor compound without a significant presence of any other species. In addition to the inhibitor compound, source B had several other compounds present which were not seen in source A inhibitor.

### *Synthetic byproducts are present in source B inhibitor compound.*

Three different columns and solvent systems were tested, as well as two different ionization sources: electrospray (ESI) and atmospheric pressure chemical ionization (APCI). APCI provided universally better signal than ESI, although it appears to lead to some spontaneous fragmentation (vide infra). The signal for [M + H] + at *m*/*z* 465 was 14 times greater with APCI despite loss of approximately 40% of the signal due to spontaneous fragmentation. The column and solvent system described herein yielded the best separation, with minimal chromatographic aberrations induced by the sample solvent. Several significant species were observed in the sample from source B, including CHIR99021; three of these were observed also in the sample from source A (Fig. 3). Details of the observed species are given in Table 2; note that while peak areas are reported in the absence of standards the impurities cannot be quantified with accuracy, and no attempt is made to do so. Based on the parent ions observed and the fragment ion spectra of those ions, possible identities for some of the impurities are discussed below.

**Table 2:** Significant chemical species observed in LCMS data for CHIR99021 from source B

RT (min)	Species	Ions observed (*m*/*z*)	No. of Cl atoms	Diagnostic fragments (*m*/*z*)	Peak area at 340 nm, ×10^3^
23.60	A	296/298	2	173/175	2.6
24.95	B	321	0	146	21.6
25.42	C	320/322	2	–	1.4
27.75	D	321/323	2	175, 147, 120	36.7
32.95	E	465/467	2	146	1742
34.35	F	479/481	2	146	114.8
35.90	G	466/468	2	146	23.7
38.80	H_1_	429/431	1	146	27.1
39.15	H_2_	429/431	1	146	17.7

### *Species A through D.*

**Figure 4.**
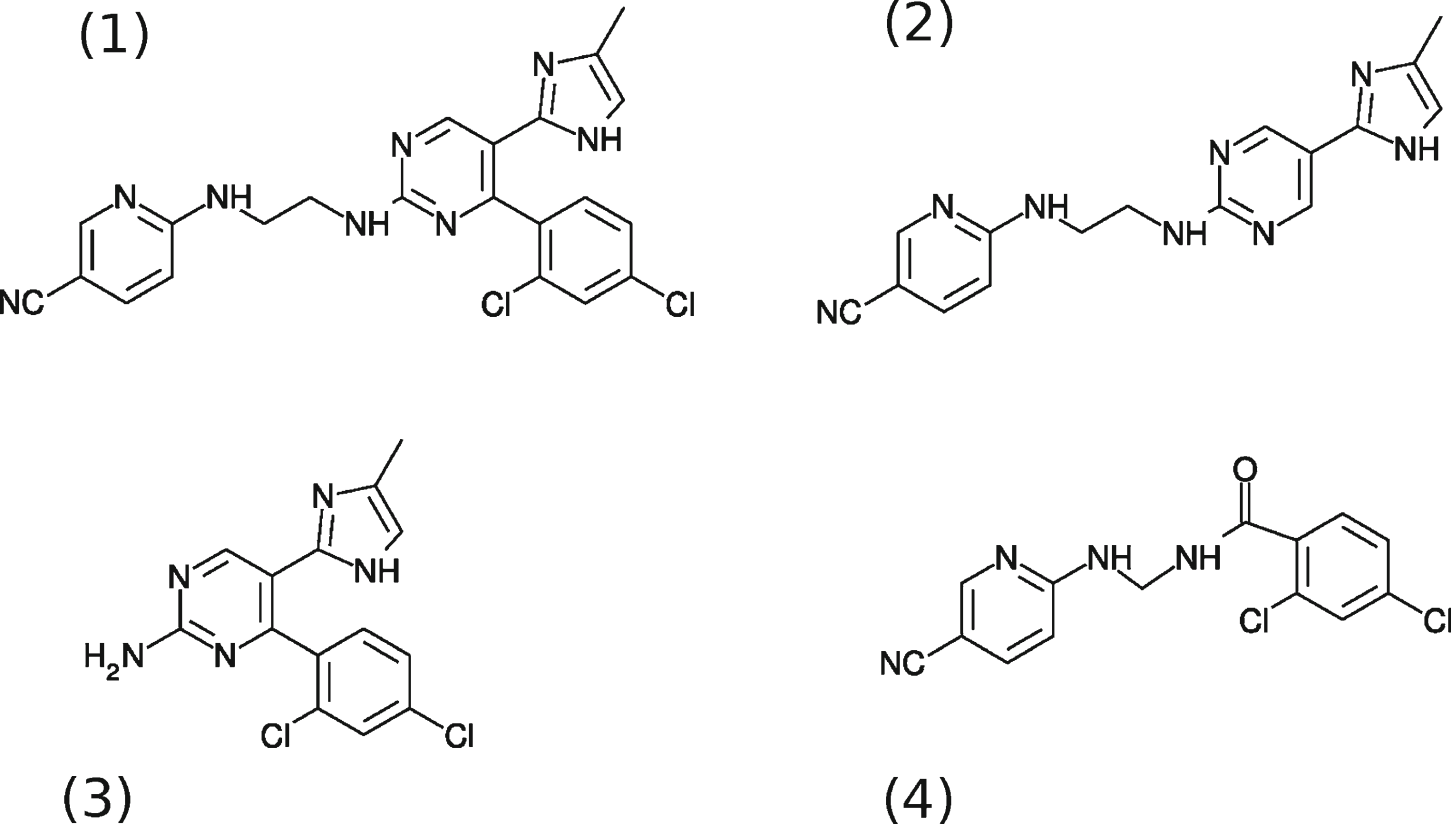
Structures for some of the observed species: (*1*) CHIR99021, (*2*) species B, (*3*) species C, and (*4*) species D.

For species A, the dominant fragments are at *m*/*z* 173/175 with an isotope pattern indicating two chlorine atoms, which is a 2,4-dichlorophenylcarbonyl moiety; the data is insufficient to conclude anything other than that this species arises from improper linkage or possibly inadequate cleanup of products early in the synthesis. For species B, a fragment at *m*/*z* 146 is present (arising from the cyanopyridyl end of the molecule), but chlorine is absent. The mass difference between this and CHIR99021 can be explained by absence of the dichlorophenyl moiety. In *2* of Fig. 4, the postulated structure was shown. For species C, the diagnostic fragment at *m*/*z* 146 is absent, indicating an absence of the cyanopyridyl end of the molecule; the mass difference is consistent with such an absence, and the structure is shown in *3* of Fig. 4. For species D, the most abundant fragment ion is *m*/*z* 175, arising from loss of neutral dichlorobenzene from the parent ion; this is followed by neutral loss of CO and, subsequently, HCN to give a fragment at *m*/*z* 120. Based on this, we theorize the structure in *4* of Fig. 4 for this analyte.

### *Species E–H.*

Species E shown in *1* of Fig. 4 is intact CHIR99021. It appears to undergo a significant degree of in-source fragmentation due to the temperature and/or chemistry of the ion source; observed fragments in the LCMS data include *m*/*z* 429/431 (loss of HCl), *m*/*z* 320/322 (loss of the cyanopyridyl moiety), *m*/*z* 310/312 (loss of cyanopyridylamine from the fragment at *m*/*z* 429/431), *m*/*z* 284/286 (loss of HCl from the fragment at *m*/*z* 320/322), and *m*/*z* 146 (a cyanopyridylaminoethyl group, which turns out to be a diagnostic fragment for the presence of this moiety). Species F is greater in mass than CHIR99021 by 14 Da but has a very similar fragmentation pattern and appears to be identical to CHIR99021 save for an extra methylene in an undetermined location on the dichlorophenyl end of the molecule. Species G yields the diagnostic fragment at *m*/*z* 146 (inclusion of the cyanopyridyl moiety) but is otherwise of uncertain structure. Species H_1_ and H_2_ have the same fragment ion spectra, which are identical to the fragmentation of CHIR99021 following its initial loss of HCl; these apparently isomeric species seem to arise from condensed phase loss of HCl whether during the synthesis or storage is uncertain.

There are numerous other less abundant species present, most being too weak for reliable MS/MS but some having the recognizable chlorine isotope pattern and the fragment ion at *m*/*z* 146 which indicates the presence of the cyanopyridyl group. Although most of the impurities are not identified and none of them are quantified, the important observation is that various impurities structurally similar to CHIR99021 are present and are believed to give rise to chromosomal abnormalities in the ES cell culture experiments.

## Discussion

The derivation of rat ES cells has become an important tool in the application of gene targeting and related technologies for many aspects of biomedical research (Buehr et al. 2008). Rat ES cell culture is carried out routinely in various laboratories. After derivation of a rat ES cell line, cells are passaged to expand the line and also to avoid differentiation by overcrowding of ES colonies. It is well-established that ES cells from many species, including rat, are reasonably stable at early passage numbers, but chromosomal abnormalities increase with higher passages (Liu et al. 1997; Li et al. 2008; Narva et al. 2010). If injected into blastocysts, these ES cells frequently result in chimeric animals that do not demonstrate germline transmission of the genetic contribution of the ES cells (Fedorov et al. 1997; Longo et al. 1997). In our study, we show that by using CHIR99021 from source B, the number of aneuploid cells increased considerably in three different rat ES cell lines even at lower passages.

Rat media consists of several components each having unique and important functions in maintaining pluripotency and overall viability with cell growth (Ying et al. 2008). To sustain efficient ES cell self-renewal, CHIR99021 inhibitor (Ying et al. 2008) is used with PD0325901 inhibitor (Bain et al. 2007) at very specific concentrations in ES cell media. A higher dose of PD0325901 causes suppression of ERK activation causing growth arrest and cell death but with addition of CHIR99021 which down-modulates Gsk3; the combination of these two inhibitors maintains metabolic activity, biosynthetic capacity, and overall viability (Ying et al. 2008). This crucial role of inhibitors shows that concentrations of individual components in ES cell media are critical to maintain ES cell characteristics. Any change in their concentrations can cause unwanted side effects in ES cell culture.

Genome stability requires that replicated chromosomes are accurately segregated during mitosis (Nicklas 1997). Chromosome segregation is facilitated by a microtubule spindle, where chromosomes attach via their kinetochores, complex microtubule-binding structures which assemble at the centromeric heterochromatin (Cleveland et al. 2003; Maiato et al. 2004; Chan et al. 2005). Cells treated with Gsk3 inhibitors frequently show abnormal spindle morphology with elevated numbers of astral microtubules, and thus in cultured cells, Gsk3 inhibitors induce chromosome non-disjunction which may cause unexpected induction of chromosome instability (Tighe et al. 2007).

In our study, we found that three different rat ES cell lines derived from different rat strains/stocks and at different passage numbers (relatively low for SD.Tg.EC1 and F344.Tg.EC4011 while relatively high for DA-EC8) showed greatly increased aneuploidy when treated with source B CHIR99021 inhibitor while remaining relatively stable in the presence of source A CHIR99021.

Based on the abnormal karyotypes seen, it is possible that many of the abnormal cells are showing accumulation of non-disjunction events in each successive passage, for example, in the F344-Tg.EC4011 cell line grown in media containing source B inhibitor, monosomy 13 was seen in 29% of the abnormal cells with other abnormal cells showing loss of another Chromosome (10 or 15) in addition to loss of Chromosome 13. Loss of Chromosome 13 was the most common abnormality for both F344-Tg.EC4011 and DA-EC8 regardless of the source of inhibitor. Although no single Chromosome was selectively lost in the cells from all three different cell lines, loss or gain of Chromosomes 19 was found in all three. Observations of karyotypic abnormalities preferentially involving a particular Chromosome have been noted in mouse ES cells, where, in surveys of mouse ES cell lines, trisomy 8 often predominates (Liu et al. 1997; Sugawara et al. 2006).

Interestingly, SD.Tg.EC1 seemed particularly prone to Chromosome 14 monosomy. This cell line was derived from the SD-Tg(UBC-EGFP)2BalRrrc (RRRC#065) rat strain and is hemizygous for a single EGFP transgene whose insertion site is on Chromosome 14 (Bryda et al. 2006; Men et al. 2012). The fact that Chromosome 14 seemed to be selectively lost suggests the presence of the transgene may impact proper chromosomal segregation, especially under conditions (increased Gsk3 inhibition) that are leading to increased non-disjunction.

The LCMS analysis of source B inhibitor showed the presence of small synthetic molecules which resemble byproducts of synthesis and/or purification of CHIR99021 and also fragments of the main inhibitor molecule. We speculate that the presence of these byproducts actually results in increased bioactivity of this compound. In essence, the amount of inhibitor is greater than expected which leads to an increase in downstream effects on chromosome stability. This, in turn, increases the level of aneuploidy in ES cell culture. Additionally, it has been reported recently that when the amount of Gsk3 inhibitor CHIR99021 was decreased from 3.0 to 1 μM in the media of cultured rat embryonic stem cells (ESCs), less differentiation occurred (Chen et al. 2013). Therefore, unrecognized elevated bioactivity in CHIR99021 from different vendor sources could also negatively impact rat ESC cell self-renewal and increase unwanted differentiation.

## Conclusion

Rat ES cell culture media greatly influences the characteristics of rat ES cells and one important characteristic necessary for successful use of ES cells for genetic manipulation is the presence of normal karyotypes in the majority of the cells. While conducting routine rat ES cell culture in our lab, we observed increased levels of aneuploidy in rat ES cells when the media contained source B inhibitor which we chose over the previous source A inhibitor for its comparable purity and lower cost. Our analysis of LCMS experiments showed the presence of small synthetic molecules in source B inhibitor which are absent in source A inhibitor. We suspect that these small synthetic molecule species are a result of either the synthesis and/or purification process. We speculate that the presence of these synthetic molecules alters the effective concentration of bioactive Gsk3 inhibitor, contributing to the higher aneuploidy levels in rat ES cells. This study is a cautionary tale emphasizing the importance of documenting the source of all reagents and carefully assessing their impact by routine quality control measures.
